# Special Issue “Hybrid Drugs: Design and Applications”

**DOI:** 10.3390/ph16101358

**Published:** 2023-09-26

**Authors:** Parvesh Singh, Vipan Kumar

**Affiliations:** 1 School of Chemistry and Physics, University of KwaZulu-Natal, P/Bag X54001, Westville, Durban 4000, South Africa; 2 Department of Chemistry, Guru Nanak Dev University, Amritsar 143005, India

The widely held belief in the potential superiority of agents capable of modulating multiple biological targets has led to the adoption of molecular hybridization as an effective technique in the realm of drug discovery and development. This approach aims to amalgamate two or more pharmacophoric or bioactive subunits into a novel chemical entity, referred to as a hybrid molecule. In comparison to their parent drugs, these newly created hybrids often exhibit better affinity and efficacy, an altered selectivity profile characterized by improved pharmacokinetic and pharmacodynamic properties, the ability to engage in dual or multiple modes of action, diminished undesirable side effects, decreased instances of drug–drug interactions, reduced likelihoods of drug resistance emergence or proliferation in pathogens, and enhanced cost-effectiveness [1,2,3,4,5,6,7]. Our invitation for submissions to this Special Issue generated significant interest from a diverse spectrum of researchers, resulting in the inclusion of four research papers and four review articles.

Gustavo Moreno-Quintero and collaborators conducted a study highlighting the promising chemopreventive potential of a series of hybrid molecules combining 5-fluorouracil (5-FU) and genistein and evaluating these hybrids on human colon adenocarcinoma cells (SW480 and SW620) as well as non-malignant cell lines (HaCaT and CHO-K1). Notably, these hybrids induced cell cycle arrest predominantly at the S-phase and G2/M checkpoints. Moreover, the investigation revealed that the most potent hybrids induced apoptosis in SW620 cells, implying the engagement of the extrinsic pathway. This apoptotic reaction was closely associated with the activation of p53, substantiated by heightened levels of caspases 3/8 and the tumor suppressor protein Tp53 [8].

Aida Šermukšnyt˙e conducted a study in which a series of hydrazones were synthesized by reacting 1,2,4-triazol-3-ylthioacetohydrazide with isatins. The synthesized compounds were assessed for their cytotoxic effects on various cancer cell lines, including human melanoma IGR39, human triple-negative breast cancer (MDA-MB-231), and pancreatic carcinoma (Panc-1) cell lines. Although these derivatives did not exhibit substantial selectivity towards cancer cells compared to fibroblasts, they demonstrated heightened cytotoxicity against the triple-negative breast cancer cell line [9].

Simona Cavalu and her collaborators showcased the promising prospects of integrating 1,3,4-oxadiazole compounds with other established anticancer pharmacophores. The central objective of their review was to underscore the anticancer potential of 1,3,4-oxadiazole derivatives. Notably, the primary emphasis was placed on the inhibition of specific biological targets critical to cancer progression. These targets included telomerase activity, HDAC, thymidylate synthase, and the thymidine phosphorylase enzyme [10].

In a recent study by Pradeep Kumar et al., a compilation of data spanning a decade (2011–2021) shed light on a plethora of hybrid antiproliferative and antitumor agents. Collectively, this comprehensive review serves as a testament to the immense potential of merging distinct pharmacophoric subunits from diverse chemical prototypes [11].

A comprehensive review authored by Wissal Liman and collaborators centered on elucidating the prevalent techniques employed for the design and synthesis of hybrid molecules. While significant strides have been taken in synthesizing potential hybrid anti-HIV drugs, it is pertinent to acknowledge that no such drug has yet progressed to the development stage or preclinical trials. While these synthesized hybrid drugs have demonstrated encouraging outcomes in vitro, they have regrettably exhibited limited in vivo activity. These obstacles necessitate in-depth exploration and investigation to devise novel hybrid anti-HIV drugs capable of surmounting these impediments and attaining optimal efficacy [5].

Najeeb Ur Rehman and colleagues published a study unveiling a series of 1H-1,2,3-triazole hybrids derived from 3-*O*-acetyl-11-keto-β-boswellic acid (β-AKBA) and 11-keto-β-boswellic acid (β-KBA) through the utilization of click chemistry. Their research delved into α-glucosidase inhibition, revealing that all the synthesized derivatives exhibited remarkable potency, exhibiting IC_50_ values ranging from 0.22 to 5.32 μM. Notably, these compounds surpassed the efficacy of the standard acarbose by several-fold [12].

Cele and their research team published findings regarding the inhibitory effects on α-glucosidase and α-amylase, as well as the antioxidant properties of quinoline hybrids incorporating 1,3,4-oxadiazole and 1,2,3-triazole cores. Among these compounds, the compound featuring a bromopentyl side-chain demonstrated the most potent α-glucosidase inhibition (IC_50_ = 15.85 μM), outperforming the reference drug acarbose (IC_50_ = 17.85 μM). Two of the compounds emerged as highly effective NO scavengers (IC_50_ = 2.67 and 3.01 μM, respectively), surpassing the performance of gallic acid (IC_50_ = 728.68 μM) [13].

Chowdhary and co-workers explored the potential of isatin-based hybrid compounds as a strategy to combat drug resistance while offering novel compounds with diverse mechanisms of action and favorable safety profiles. In the context of antimycobacterial agents, the incorporation of isatin alongside isoniazid not only enhanced the lipophilicity and activity of these hybrids but also reduced the likelihood of resistance development. Combining the isatin core with the quinoline core yielded hybrids with superior antiplasmodial properties compared to chloroquine (CQ) itself, particularly against CQ-resistant strains of *P. falciparum*. The existing evidence strongly suggests that further harnessing the potential of isatin-based compounds could yield effective clinical candidates with a lower risk of drug resistance emergence [14].

In conclusion, hybridization protocol has emerged as a powerful asset for the development of more potent and less toxic drugs that exhibit increased specificity compared to conventional mono-therapy. There is substantial potential in refining and exploring novel designs, utilizing linker structures, and meticulously choosing partners and functional entities. These endeavours offer the prospect of discovering new drug candidates that could outperform existing anti-infective and anticancer medications in terms of safety and efficacy. Notably, numerous hybrid molecules have advanced through both early and late phases of clinical development, highlighting the promising role of hybridization as an innovative approach to drug discovery.

## Abbreviations

5-FU, 5-fluorouracil; β-AKBA, 3-*O*-acetyl-11-keto-β-boswellic acid; β-KBA, 11-keto-β-boswellic acid; IC_50_, half-maximal inhibitory concentration; CQ, chloroquine; NO, nitric oxide; *Plasmodium falciparum*, *P. falciparum*.
